# N-*acyl*-homoserine lactonase–nanosilver composite modulates the bacterial community of an *in vitro* enriched biofilm model of early childhood caries

**DOI:** 10.3389/froh.2026.1779148

**Published:** 2026-10-02

**Authors:** Chuang Nie, Ran Xia, Liqiong Ma, Song Li, Jing Zhang

**Affiliations:** 1 Anhui Provincial Key Laboratory of Oral Diseases Research, College & Hospital of Stomatology, Anhui Medical University, Hefei, China; 2 Faculty of Dentistry, The University of Hong Kong, Hong Kong, Hong Kong SAR, China

**Keywords:** biofilms, early childhood caries, N-*acyl*-homoserine lactones, quorum-quenching enzyme, silver nanoparticles

## Abstract

**Objectives:**

This study investigated the effects of a nanocomposite comprising *N-acyl*-homoserine lactonase Est816 and silver nanoparticles (Ag-Est816) on the microbial community of an enriched *in vitro* biofilm model derived from supragingival plaque of children with early childhood caries (ECC).

**Materials and methods:**

Supragingival plaque samples were collected from 30 children aged younger than 6 years (15 males and 15 females) diagnosed with ECC. These samples were cultured *in vitro* to form biofilms, which were then treated with or without the Ag-Est816 composite for two days. The composition of the biofilms was analyzed by 16S rRNA sequencing to assess changes in *α*- and *β*-diversity, taxonomic composition, and predicted microbial functions. Biofilm morphology and biomass were evaluated using scanning electron microscopy (SEM) and crystal violet assays.

**Results:**

The Ag-Est816 composite significantly reduced microbial *α*-diversity, including species richness and phylogenetic diversity (Chao1, Observed species, Faith's PD, *p* < 0.05). It also significantly altered overall bacterial community composition, as demonstrated by principal coordinate analysis (PCoA) and paired permutational multivariate analysis of variance (PERMANOVA), although the effect size was modest (*R*^2^ = 0.0286, *P* = 0.002). At the genus level, the abundances of *Actinomyces*, *Lactobacillus*, and *Moryella* were markedly decreased, whereas *Haemophilus* and *Granulicatella* were enriched. LEfSe analysis revealed *Actinomyces* and *Lactobacillus* as characteristic taxa enriched in the Control group, while *Enterococcus* and *Granulicatella* were significantly enriched following treatment. Functional prediction revealed that Ag-Est816 influenced pathways related to carbohydrate metabolism, amino acid biosynthesis, nucleotide metabolism, and nucleotide sugar biosynthesis. SEM imaging and biomass quantification confirmed that the Ag-Est816 composite disrupted biofilm architecture and significantly reduced total biomass.

**Conclusion:**

The Ag-Est816 composite suppressed several low-abundance cariogenic genera while reducing overall community richness, altered microbial metabolic functions, and compromised biofilm structural integrity. These findings indicated this non-invasive nanocomposite has potential as an adjunctive therapy for caries prevention in children.

## 1 Introduction

Early Childhood Caries (ECC) is one of the most prevalent chronic oral diseases worldwide and poses a serious public health concern. Epidemiological studies estimate that approximately 532 million children are affected by untreated deciduous tooth caries (1). ECC can lead to the destruction of dental hard tissues, pulpal and periodontal infections, and even premature loss of primary teeth. These consequences impair masticatory function, disrupt the eruption of permanent teeth, and negatively affect craniofacial development (2). Due to the immature physiological and psychological development, children often have difficulty in cooperating during dental treatment. Dental fear and poor compliance frequently hinder the completion and effectiveness of therapy. Therefore, developing strategies to effectively inhibit and disrupt cariogenic biofilms is crucial for the prevention and management of ECC.

Silver nanoparticles (AgNPs), characterized by a high surface area and efficient silver ion (Ag⁺) release, exhibit broad-spectrum antibacterial activity against both Gram-positive and Gram-negative bacteria. AgNPs act through multiple mechanisms—including disruption of bacterial membranes, generation of reactive oxygen species, and interference with DNA replication—making the development of resistance less likely (3–5). *Streptococcus mutans*, a primary cariogenic microorganism in the oral cavity, is particularly susceptible to AgNPs, which have demonstrated stronger inhibitory effects than silver nitrate or silver diamine fluoride (6, 7). A clinical randomized controlled trial further confirmed that combining AgNPs with fluoride significantly reduced the activity of dentin caries in primary molars (8). However, challenges remain in applying AgNPs for caries management. AgNPs tend to aggregate, reducing their antibacterial efficacy (9); certain silver-based formulations may cause tooth or restorations discoloration (10); and prolonged or high-concentration exposure to silver ions may exert cytotoxic effects on dental pulp stem cells and odontoblasts (11, 12). Therefore, developing safer and more controlled application strategies is essential.

Biofilm process is highly regulated by quorum sensing, an intercellular communication mechanism mediated by signaling molecules. Among these, *N-acyl*-homoserine lactones (AHLs) are typical quorum sensing signals in Gram-negative bacteria. The accumulation of AHLs allows bacteria to sense population density and coordinate collective behaviors, including biofilm formation, virulence factor production, and antibiotic resistance gene expression (13). Although AHLs are primarily produced by Gram-negative species, Gram-positive bacteria can also respond to AHLs signaling, suggesting that AHLs mediate interspecies communication within complex microbial communities (14). Therefore, inhibiting AHL-mediated quorum sensing represents a promising and non-bactericidal strategy.

Approaches to inhibit AHLs signaling include the use of AHLs synthesis inhibitors, competitive antagonists, and AHLs-degrading enzymes. Among these, enzymatic degraders have gained increasing attention due to their high substrate specificity, low cytotoxicity, and strong environmental stability, as well as their ability to interfere with quorum sensing without affecting bacterial growth or metabolism. Aii20J, a broad-spectrum AHL-lactonase, has been reported to significantly inhibit biofilm formation in both healthy individuals and patients with periodontitis. Moreover, it exhibits potent inhibitory effects on mixed biofilms composed of six oral pathogenic bacteria (15).

In our previous work, we identified a novel AHL-lactonase, Est816, with high hydrolytic activity against all types of medium- and long-chain AHLs. Est816 has excellent stability at pH: 5.0–9.0 and below 50 °C, which is suitable for oral application with good biocompatibility (16). Additionally, we developed an innovative antimicrobial composite by covalently conjugating Est816 to AgNPs via branched polyethyleneimine, termed Ag-Est816 (17). This dual-function composite aims to exert synergistic effects by simultaneously disrupting quorum sensing signaling and providing direct bactericidal activity as well. In this study, we aimed to evaluate the effect of Ag-Est816 on the bacterial community of an ECC-derived enriched *in vitro* biofilm model established from plaque samples of 30 children with ECC using 16S rRNA gene high-throughput sequencing. The findings are expected to provide theoretical and experimental support for the development of novel ecological intervention strategies for the prevention and management of pediatric dental caries.

## 2 Materials and methods

### 2.1 Preparation of composite

The Ag-Est816 composite was prepared according to our previous study. In brief, 25.5 mg of BPEI (branched polyethylenimine) (Mw 40k) was dissolved in 16.25 mL of deionized water and added to a 100 mL ﬂask. An 8.75 mL lactonase solution (30 U/mL) was added to the flask and stirred for 30 min, followed by dropwise addition of 12.5 mL silver nitrate solution (0.68 mg/mL) and water (18). Then the mixture reacted for 3 h at 25 °C under magnetic stirring.

### 2.2 Ethical approval of studies

This study was approved by the Medical Ethics Committee of the Affiliated Stomatological Hospital of Anhui Medical University, China (Protocol No. JY2025008-A).

### 2.3 Participant selection

Between April 2025 and October 2025, a total of 30 children younger than 6 years were recruited from the Department of Pediatric Dentistry at the Affiliated Stomatological Hospital of Anhui Medical University. Dental plaque samples were aseptically collected from carious lesions using sterile cotton swabs. Inclusion criteria were as follows: (1) younger than 6 years; (2) presence of at least one primary tooth with active caries; and (3) informed consent obtained from the child and their guardian. Exclusion criteria included: (1) extensively decayed teeth that had already been restored; (2) carious surfaces that were smooth and hard; (3) presence of hereditary or systemic diseases; (4) history of long-term medication or antibiotic use within the past 3 months; and (5) topical fluoride treatment within the past 2 weeks.

### 2.4 Dental plaque collection

The sampling area was first rinsed with sterile saline and isolated using sterile cotton rolls to maintain dryness. Sterile cotton swabs were then used to collect plaque from carious sites. Each sampling procedure involved gently scraping the lesion area back and forth 3–4 times. A separate sterile cotton swabs was used for each sample to assist in plaque collection. The tip of the swab containing the dental plaque was immediately immersed in 1 mL sterile phosphate-buffered saline (PBS) for 4–5 s. After sealing the collection tube, the sample was vortexed for 4–5 s to maximize dispersion of the plaque material. All collected samples were labeled with a unique code and cultured in brain heart infusion (BHI) broth through two successive rounds of BHI-based amplification. The resulting enriched cultures were then used as inocula to establish biofilms under aerobic conditions, generating an enriched *in vitro* biofilm model. Following incubation, the cultures were centrifuged at 4,000 rpm for 10 min to remove the supernatant. The bacterial pellet was then mixed with 1 mL bacterial cryopreservation solution, rapidly frozen at −80 °C, and labeled accordingly. Sample labels included the sample ID, patient's gender and age, and the tooth position from which the plaque was collected.

### 2.5 Biofilm formation and treatment with the Ag-Est816 composite

Frozen plaque samples were rapidly thawed, and 10 μL of each bacterial suspension was inoculated into 2 mL of BHI in a sterile centrifuge tube, as a pre-culture step to amplify the limited biomass obtained from ECC-derived plaque samples to a standardized level suitable for downstream biofilm construction and molecular analyses. The cultures were incubated aerobically at 37 °C for 48 h. After incubation, the bacterial suspension was centrifuged at 4,000 rpm for 10 min to remove the supernatant. The bacterial pellet was then resuspended in 5 mL of fresh BHI and cultured again under the same aerobic conditions for 48 h to further standardize the bacterial biomass for reproducible biofilm formation. Before seeding, the bacterial suspension was adjusted to an optical density at 600 nm (OD600) of 0.2. Sterile round glass coverslips (9 mm in diameter) were placed in 24-well culture plates in advance and sterilized under UV light for 30 min. For biofilm formation, 200 μL of the standardized bacterial suspension was added to each well. In the experimental group, 200 μL of Ag-Est816 composite solution was added, while the Control group received 200 μL of PBS. In our previous studies, results indicated that synergistic biofilm disruption could be achieved at an enzyme activity of 6 U/mL and a silver concentration of 5 μg/mL, while simultaneously retaining >90% of the quorum-quenching enzyme activity and demonstrating excellent cytocompatibility with human gingival fibroblasts (17). Consequently, in the present study, a silver concentration of 5 μg/mL and an enzyme activity of 6 U/mL were selected as the final working concentrations. After gentle mixing, the plates were incubated aerobically at 37 °C for 48 h to allow biofilm formation.

### 2.6 Laboratory processing

The biofilms were gently washed three times with sterile PBS to remove loosely attached cells. The remaining biofilm-associated bacteria were then detached by vigorous pipetting (20 times) using a 1 mL pipette tip with sterile PBS. The resulting bacterial suspension was collected into sterile 1.5 mL microcentrifuge tubes. Subsequently, a secondary rinse of the biofilm surface was carried out with 500 μL PBS, and the rinse was combined with the initial suspension to maximize biomass recovery. Samples were centrifuged at 4,000×*g* for 10 min at 4 °C to pellet the bacterial cells. The supernatant was carefully discarded, and the pellet was resuspended in 200 μL of sterile PBS containing a nuclease inhibitor and stored at −80 °C until further use. Processed samples were transported on dry ice to Personalbio (Shanghai, China) for laboratory procedures.

### 2.7 DNA extraction

Frozen samples were removed from storage, and approximately 0.2 g of each sample was quickly transferred into centrifuge tubes containing extraction lysis buffer for homogenization. Sample grinding was performed using a Tissuelyser-48 multi-sample tissue grinder (Jingxin, Shanghai, China) at a frequency of 60 Hz. Genomic DNA was extracted using the MagBeads FastDNA Kit for Soil (116564384; MP Biomedicals, CA, USA) according to the manufacturer's instructions. The quality and molecular size of the extracted DNA were evaluated by 0.8% agarose gel electrophoresis, and DNA concentration was quantified using a NanoDrop spectrophotometer. Only samples passing quality control criteria (adequate integrity and purity) were included in subsequent 16S rRNA sequencing. All 60 samples passed DNA quality control, and no samples were excluded, resulting in 30 paired samples (60 samples in total) for downstream analysis.

### 2.8 Sequencing analysis

The V3–V4 hypervariable regions of the bacterial 16S rRNA gene were amplified using the primer pair 338F (5′-ACTCCTACGGGAGGCAGCA-3′) and 806R(5′-GGACTACHVGGGTWTCTAAT-3′). PCR reactions were performed in triplicate for each sample using a high-fidelity DNA polymerase to minimize amplification bias. PCR cycling conditions were: initial denaturation at 98 °C for 5 min; 26 cycles of 98 °C for 30 s, 52 °C for 30 s, and 72 °C for 45 s; and a final extension at 72 °C for 5 min. Amplicon purified amplicons were performed on NovaSeq 6000 (Illumina Company, San Diego, CA, USA). Before sequencing, all libraries with non-redundant index sequences were normalized and diluted to 2 nM, followed by pooling based on the desired sequencing depth for each sample. The pooled libraries were subsequently denatured into single-stranded DNA using 0.1 N NaOH prior to sequencing. Bioinformatics analysis was conducted using QIIME2 2,024.5 with slight modification according to the official tutorials. Raw sequences data were demultiplexed using the demux plugin followed by primers cutting with cutadapt plugin. Sequences were then quality filtered, denoised, merged and chimera removed using the DADA2 plugin with default parameters. The raw datasets were processed with DADA2 to generate amplicon sequence variants (ASVs). Read counts retained at each major step of the DADA2 workflow, including quality filtering, denoising, paired-end merging, and chimera removal, were recorded for each sample. The final sequencing depth was defined as the number of reads retained in the final ASV abundance table after singleton removal, and the number of observed ASVs was calculated for each sample. The sequencing quality-control results, including per-sample read counts and observed ASVs, are summarized in Supplementary Table S1. The ASV table was rarefied to 29,633 sequences per sample for alpha and beta diversity analyses; this value represents the rarefaction depth used for downstream diversity analyses rather than the original sequencing depth of individual samples.

Negative controls were included throughout DNA extraction and PCR amplification to monitor potential contamination. For the extraction blank control, no biological material was added, while all reagents were processed following the standard extraction protocol. The resulting extraction blank was further checked by agarose gel electrophoresis. For the PCR negative control (no-template control, NTC), sterile nuclease-free water was used instead of template DNA and subjected to the same amplification conditions as the samples.

### 2.9 Biofilm characterization

Biofilm morphology was observed by scanning electron microscopy (SEM), and total biofilm biomass was quantified using a crystal violet assay. For SEM analysis, biofilms were first fixed with 2.5% glutaraldehyde at 4 °C for 30 min, followed by dehydration through a graded ethanol series, critical-point drying, and gold sputter-coating before imaging. Biofilm biomass was quantified via the crystal violet method: samples were fixed with methanol, stained with 2% crystal violet for 15 min, and subsequently eluted with 95% ethanol. The absorbance at 590 nm (OD590) was recorded to determine total biomass. All experiments were performed in triplicate, and data are presented as mean values.

### 2.10 Statistical analysis

Alpha diversity indices (Chao1, Observed species, Good's coverage, Faith's phylogenic diversity and Pielou's evenness indices) were compared between groups using paired Student's *t*-test (normal distribution) or Wilcoxon signed-rank test (non-normal distribution), with Benjamini–Hochberg correction for multiple testing. Beta diversity was assessed based on weighted UniFrac distances to evaluate differences in bacterial community composition between the Control and Ag-Est816 groups. Principal coordinate analysis (PCoA) based on the weighted UniFrac distance matrix was performed to visualize patterns of overall community variation. Because samples from the Control and Ag-Est816 groups were derived from the same children, permutational multivariate analysis of variance (PERMANOVA) with subject-wise restricted permutations, using Subject ID as the blocking factor, was performed to account for the paired study design. A total of 999 restricted permutations were conducted, and the pseudo-*F* statistic, *R*^2^, and permutation-based *P* value were reported. To assess whether differences in within-group dispersion could contribute to the PERMANOVA result, permutational analysis of multivariate dispersions (PERMDISP) was performed using the same weighted UniFrac distance matrix. The *F* statistic and permutation-based *P* value were reported, with *P* < 0.05 considered statistically significant. Differential abundance analysis at the ASV and genus levels was conducted using DESeq2, with Benjamini–Hochberg false discovery rate (FDR) correction applied. Adjusted *p*-values (*q* < 0.05) were considered statistically significant. Microbial functional profiles were predicted from the 16S rRNA sequencing data using Phylogenetic Investigation of Communities by Reconstruction of Unobserved States 2 (PICRUSt2) and represented as Kyoto Encyclopedia of Genes and Genomes Orthologs (KOs). Differentially abundant KOs between the paired Control and Ag-Est816 groups were identified using the limma package in *R*, with the paired sample structure incorporated into the design matrix. *P* values were adjusted using the Benjamini–Hochberg method, and KOs with an adjusted *p* < 0.05 were considered significantly different. Kyoto Encyclopedia of Genes and Genomes (KEGG) pathway enrichment analysis was subsequently performed using the enricher function in the clusterProfiler package based on a KO–pathway mapping table. The enriched pathways were visualized using dot plots, with the top 20 pathways displayed. For crystal violet biomass quantification, all 30 paired samples per group were analyzed in technical triplicates, and the mean of the technical replicates was used for statistical comparison (*n* = 30 per group). For SEM, three representative samples per group were selected for imaging. All statistical analyses were conducted using GraphPad Prism (version 8.0; GraphPad Software, USA). Statistical significance was defined as *p* < 0.05.

## 3 Results

### 3.1 Rarefaction curve

The experiments were illustrated in Figure 1. Rarefaction curves were employed to assess whether the sequencing depth was sufficient for capturing the microbial diversity in the samples. As shown in Figure 2, the curves gradually plateaued with increasing numbers of sequencing reads, indicating that the majority of microbial species within the childhood dental caries microbiota were effectively captured. This suggests that the sequencing data are both reliable and comprehensive for subsequent analyses. Per-sample sequencing depth and DADA2 quality-control results are provided in Supplementary Table S1.

**Figure 1:**
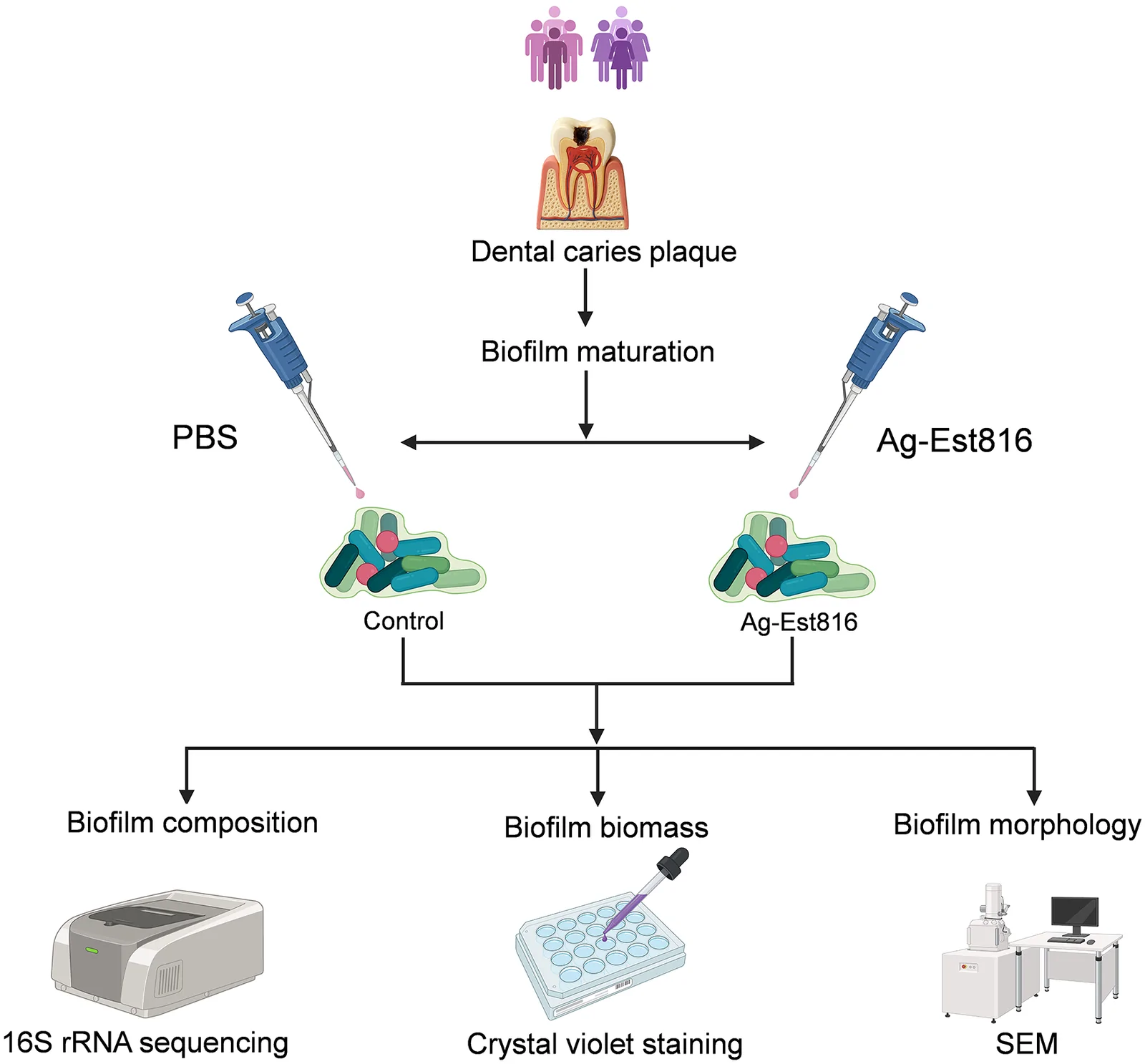
Schematic of the experimental procedure.

**Figure 2:**
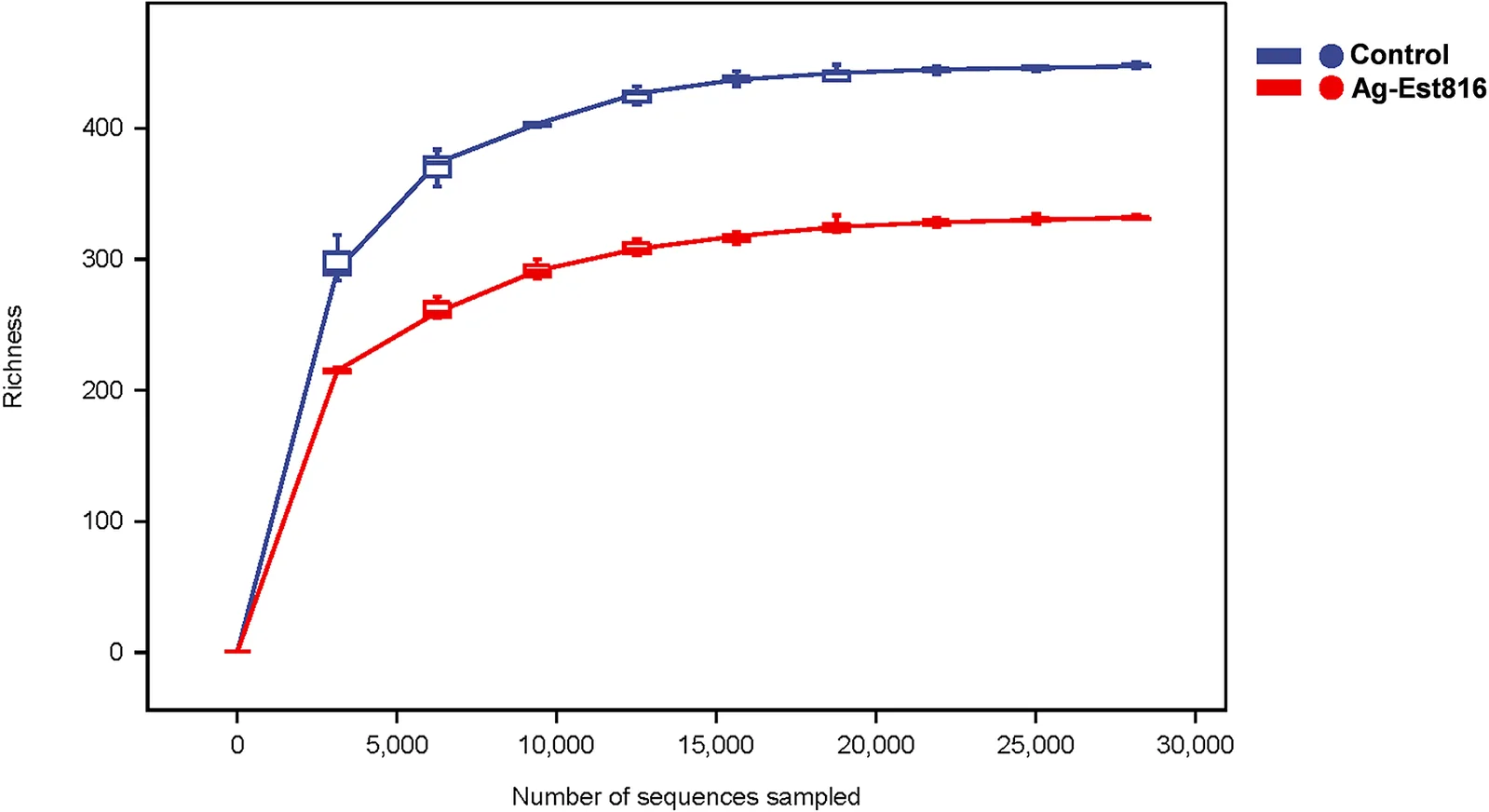
Rarefaction curves of the control and treatment groups based on the Chao1 richness index. Both groups reached saturation, indicating adequate sequencing coverage. The overall lower curve height in the treatment group suggests a reduction in microbial richness following treatment.

### 3.2 Alpha diversity

Alpha diversity was assessed using Chao1, Observed species, Good's coverage, Faith's phylogenic diversity and Pielou's evenness indices. Chao1 and Observed species reflect species richness, while Good's coverage assesses sequencing completeness. Faith's PD quantifies phylogenetic richness based on the total branch length within the phylogenetic tree. Pielou's evenness (Pielou's *E*) measures the uniformity of taxonomic distribution within the community. As shown in Figure 3, the treatment group exhibited significant decreases in Chao1, Observed species, and Faith's PD index, indicating a reduction in community richness, a lower number of detectable taxa, and overall phylogenetic diversity following treatment. In contrast, Pielou's *e* showed a slight but non-significant decline, suggesting that community evenness was largely preserved by the treatment. Good's coverage values exceeded 99% in both groups, consistent with the rarefaction curve results, confirming that the sequencing depth was sufficient to comprehensively represent the microbial communities. These results indicate that the Ag-Est816 composite significantly reduced the richness and phylogenetic diversity of this model.

**Figure 3:**
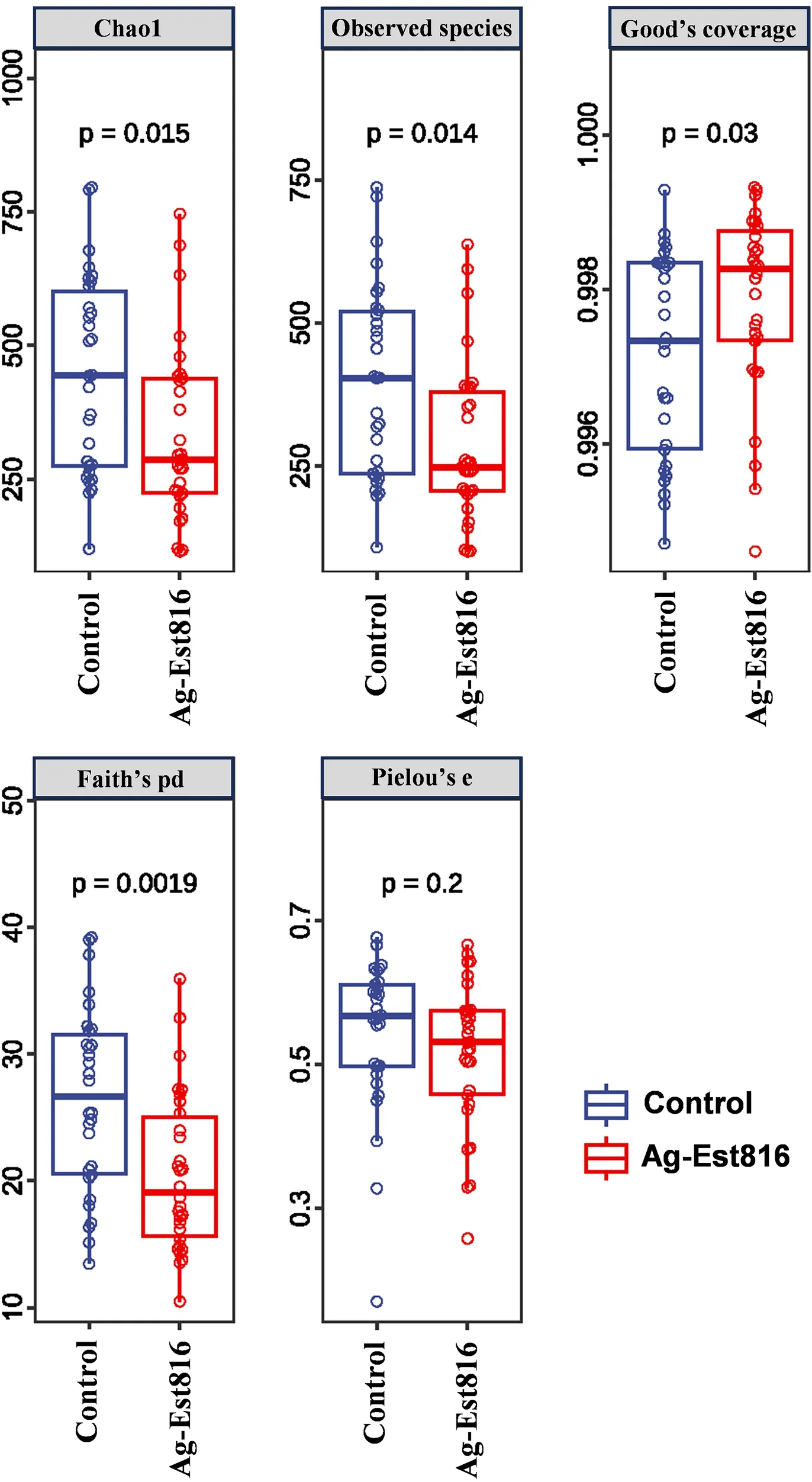
Alpha diversity of biofilm samples. The significances between control and treatment groups were evaluated using Student's *t*-test. The indices include: Richness: Chao1, Observed species; Coverage: Goods coverage; Phylogenetic diversity: Faith's PD; Evenness: Pielou's evenness.

### 3.3 Beta diversity

*β*-diversity analysis based on weighted UniFrac distance was performed to assess the differences in bacterial community composition between the Control and Ag-Est816 groups. As shown in Figure 4, PCoA revealed a separation between the two groups, with PCo1 and PCo2 explaining 42.83% and 15.93% of the variance, respectively. Considering the paired study design, samples from the same child were linked between the Control and Ag-Est816 groups in the PCoA plot.

**Figure 4:**
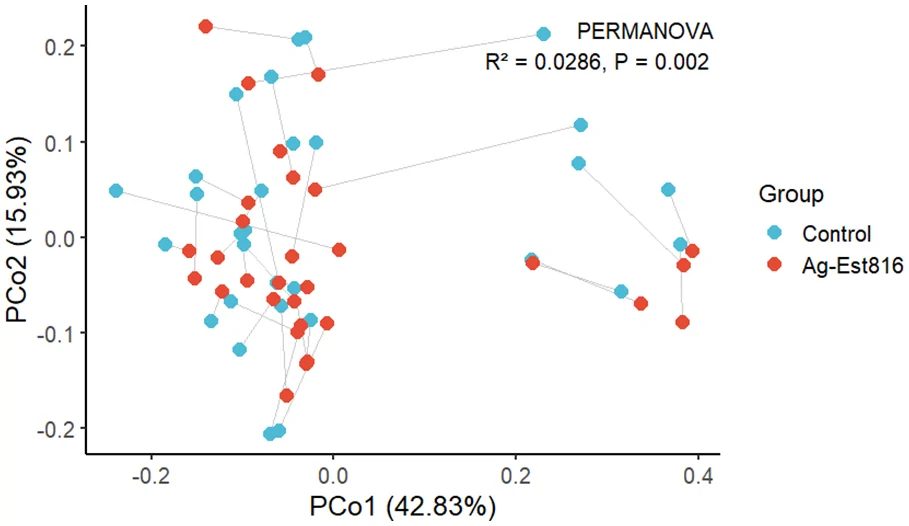
Beta diversity analysis of this model following treatment with the ag-Est816 composite. PCoA based on Weighted UniFrac distances demonstrated a clear separation of bacterial community structures between the control and Ag-Est816 treatment groups. PCo1 and PCo2 explained 47.6% and 17.7% of the total variation, respectively. The statistical significance of the difference between groups was assessed using PERMANOVA, with the corresponding *R*^2^ and *P* values shown in the figure.

Subsequently, PERMANOVA was performed, with permutations limited to within-subjects (Subject ID). PERMANOVA revealed a statistically significant but modest difference in bacterial community composition between the Control and Ag-Est816 groups (*R*^2^ = 0.0286, *F* = 1.706, *P* = 0.002; 999 subject-restricted permutations), indicating that the treatment explained approximately 2.9% of the total community variance. PERMDISP analysis detected no statistically significant difference in within-group dispersion between the two groups (*F* = 3.459, *P* = 0.064), indicating that the observed difference in community composition was not significantly associated with differences in within-group dispersion.

### 3.4 Analysis of species composition

As shown in Figure 5A, bar plots were generated to illustrate the relative abundance of bacterial phyla in each sample, ranked from highest to lowest abundance. Figure 5B presents the overall composition of bacterial phyla at the group level. Different colored bars represent different phyla, with longer segments indicating higher relative abundance. At the phylum level, a total of 13 phyla were identified based on taxonomic annotation. Phyla with a relative abundance greater than 1% were defined as dominant phyla. The dominant phyla observed across all groups included *Firmicutes*, *Proteobacteria*, *Actinobacteria*, *Fusobacteria*, and *Bacteroidetes*. Compared to the Control group, Ag-Est816 treatment exhibited a significant decrease in the relative abundance of *Actinobacteria* (7.9% in Control vs. 1.9% in Ag-Est816), while the relative abundance of *Firmicutes* (72.7% in Control vs. 80.0% in Ag-Est816) increased, as shown in Figures 5C,D.

**Figure 5:**
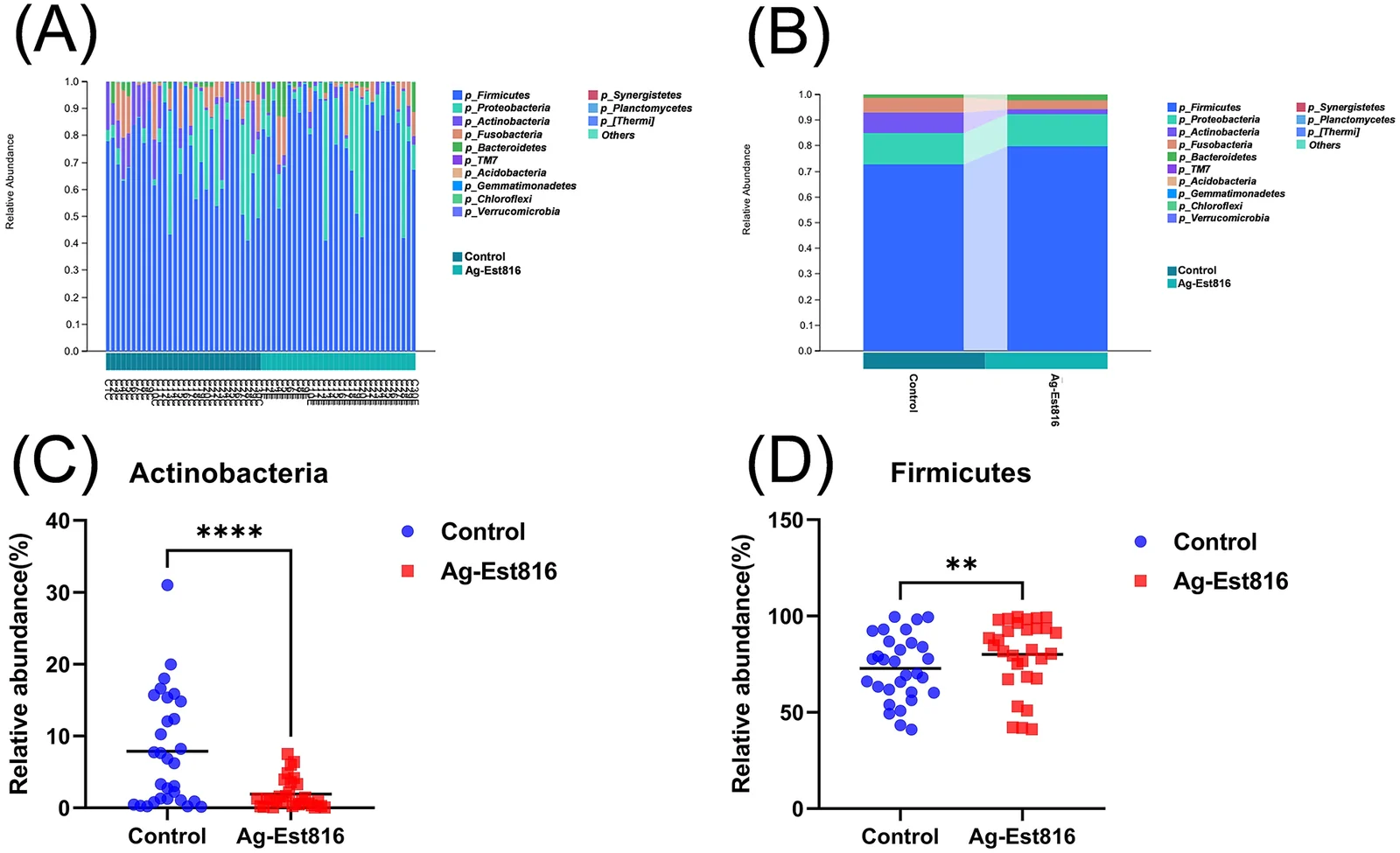
Phylum-level composition across individual samples **(A)** and per group **(B)**. Figures (C,D) illustrate the changes in selected bacterial phyla. *Actinobacteria* showed a significant decrease **(C)**, whereas *Firmicutes* exhibited an increase **(D)**.

At the genus level, the top 20 genera based on relative abundance were selected for analysis and comparison. Figure 6A illustrates the overall relative abundance of different genera across all samples. Figure 6B presents the distribution of each genus within the Control and Ag-Est816 groups. DESeq2 analysis identified significant differences in the relative abundances of several genera between the two groups. In the Control group, the dominant genera were *Veillonella* (38.1%), *Streptococcus* (25.9%), *Actinomyces* (7.1%), *Enterobacter* (6.3%), *Fusobacterium* (5.7%), *Lactobacillus* (1.7%), *Aggregatibacter* (1.5%), *Haemophilus* (1.4%), *Megasphaera* (1.3%), *Moryella* (1.3%), *Granulicatella* (1.1%), *Enterococcus* (1.1%), and *Prevotella* (1.1%). The Ag-Est816 composite significantly increased the relative abundances of *Haemophilus* (1.4% in Control vs. 2.7% in Ag-Est816) and *Granulicatella* (1.1% in Control vs. 2.1% in Ag-Est816), whereas significantly decreased the abundances of *Actinomyces* (7.1% in Control vs. 1.7% in Ag-Est816), *Lactobacillus* (1.7% in Control vs. 0.4% in Ag-Est816), and *Moryella* (1.1% in Control vs. 0.1% in Ag-Est816), as shown in Figures 6C–G.

**Figure 6:**
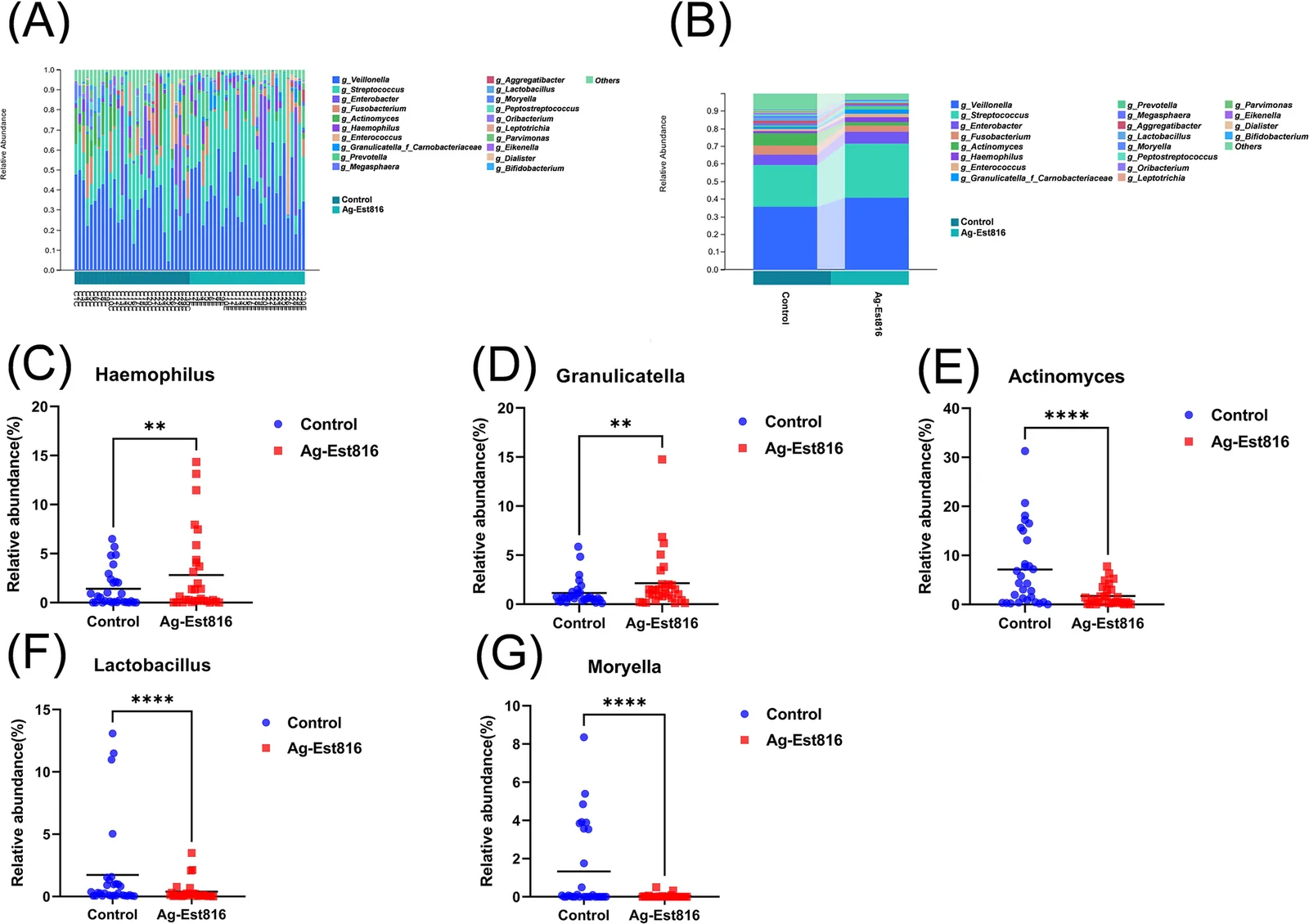
Genus-level composition across individual samples **(A)** and per group **(B)**. Figures **(C–G)** illustrate the changes in the relative abundance of selected genera. Specifically, Figures **(C,D)** show significant increases in *Haemophilus* and *Granulicatella*, whereas Figures **(E,F,G)** indicate marked decreases in *Actinomyces*, *Lactobacillus*, and *Moryella*.

### 3.5 Characteristic taxa analysis

To identify potential microbial biomarkers that distinguish the control group from the treatment group, linear discriminant analysis effect size (LEfSe) was performed. Taxa with an LDA score >2 were considered characteristic taxa. The distribution of LDA scores for taxa with significant differences is shown in the Figure 7. A total of 11 genera showing significant differences were identified between the two groups. In the Control group, the most prominent characteristic taxon was *Actinomyces* (LDA score = 4.47, *p* = 0.003). Other characteristic taxa included *Moryella* (LDA score = 3.75, *p* = 0.027), *Oribacterium* (LDA score = 3.56, *p* < 0.001), *Slackia* (LDA score = 3.16, *p* = 0.032), *Lachnoanaerobaculum* (LDA score = 3.16, *p* < 0.001), *Staphylococcus* (LDA score = 3.14, *p* = 0.038), *Campylobacter* (LDA score = 2.97, *p* = 0.024), *Catonella* (LDA score = 2.89, *p* = 0.008), and *Lactobacillus* (LDA score = 2.68, *p* = 0.004). In the treatment group, the characteristic taxa were *Enterococcus* (LDA score = 3.93, *p* = 0.007) and *Granulicatella* (LDA score = 3.76, *p* = 0.046).

**Figure 7:**
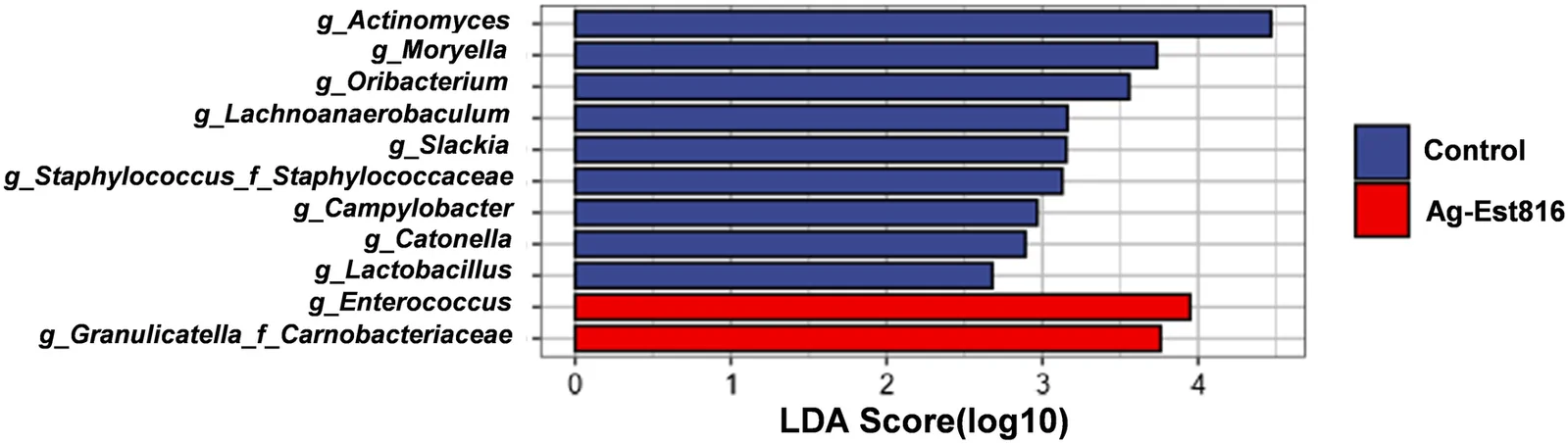
Differential bacterial taxa identified by LEfSe analysis between the control and ag-Est816. A threshold of LDA score >2 was used to define significant characteristic taxa.

### 3.6 Metabolic pathway enrichment analysis

To investigate the potential effects of the Ag-Est816 composite on microbial community functions, PICRUSt2 was used to predict significantly altered gene families, followed by KEGG pathway enrichment analysis. As shown in Figure 8, the results revealed that several key metabolic pathways were significantly enriched among the predicted differentially abundant KOs. Notably, the “Amino Acid Biosynthesis” pathway showed the most substantial enrichment, containing the highest number of KOs and exhibiting a low adjusted *p*-value. This suggests that the Ag-Est816 may have a considerable impact on amino acid metabolism. The most statistically significant alteration was observed in the “Starch and Sucrose Metabolism” pathway, indicating a strong influence of the composite on carbohydrate metabolism within the microbial community. Other significantly enriched pathways included “Nucleotide Sugar Biosynthesis” and “Purine Metabolism,” implying potential shifts in the biosynthetic and nucleotide metabolic capabilities of the plaque-associated microbiota. Additionally, the enrichment of pathways such as “Glycolysis/Gluconeogenesis”, “Fatty Acid Biosynthesis” and “Terpenoid Backbone Biosynthesis” further suggests treatment-induced changes in energy metabolism and membrane-related biosynthetic functions.

**Figure 8:**
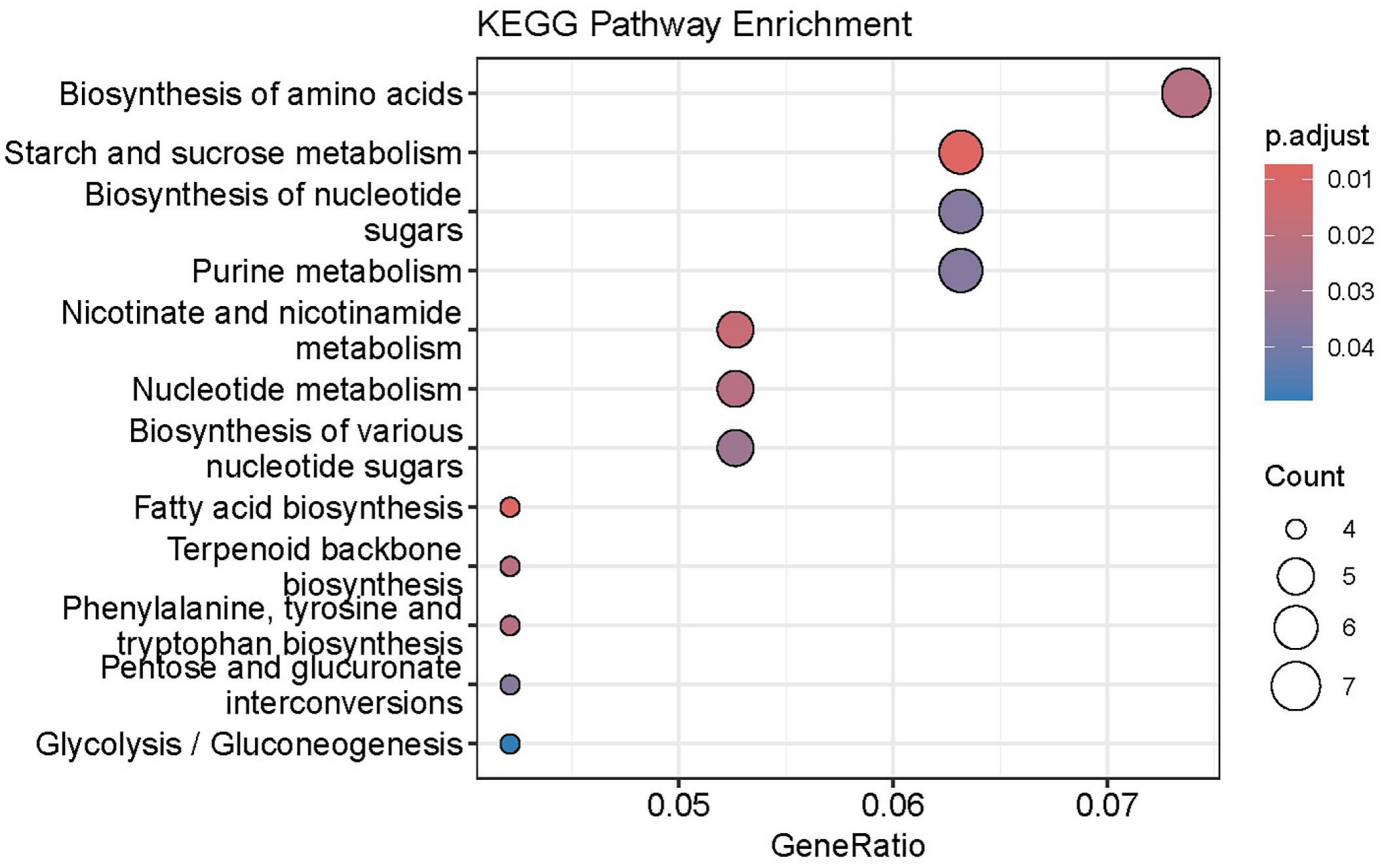
KEGG pathway enrichment analysis of predicted differentially abundant gene families. Bubble plot showing the top enriched KEGG pathways. The *x*-axis represents the GeneRatio (the proportion of genes enriched in each pathway), while the *y*-axis lists the corresponding biological pathways. Bubble size indicates the number of genes enriched in each pathway (Count), and bubble color reflects the adjusted *p*-value, with red representing more significant enrichment.

### 3.7 Ag-Est816 composite inhibited biofilms of supragingival plaque *in vitro*

To evaluate the effect of the Ag-Est816 composite on mature dental plaque biofilms associated with ECC, we analyzed the biofilm structure and biomass of all samples in both the control and treatment groups using SEM and crystal violet staining. Three representative samples were randomly selected for display, corresponding to sample No. 7 (Figures 9A–C), No.19 (Figures 9D–F) and NO.21 (Figures 9G–I). As shown in Figures 9, SEM imaging showed that biofilms exposed to the composite were more dispersed, with a substantial decrease in extracellular matrix. After crystal violet staining, microscopic examination revealed that the biofilm became markedly dispersed biofilm. Quantitative analysis further demonstrated a significant reduction in biofilm biomass in the treatment group compared with the control group. Together, these findings indicate that the composite exerts a pronounced inhibitory effect on the formation of dental plaque biofilms in children.

**Figure 9:**
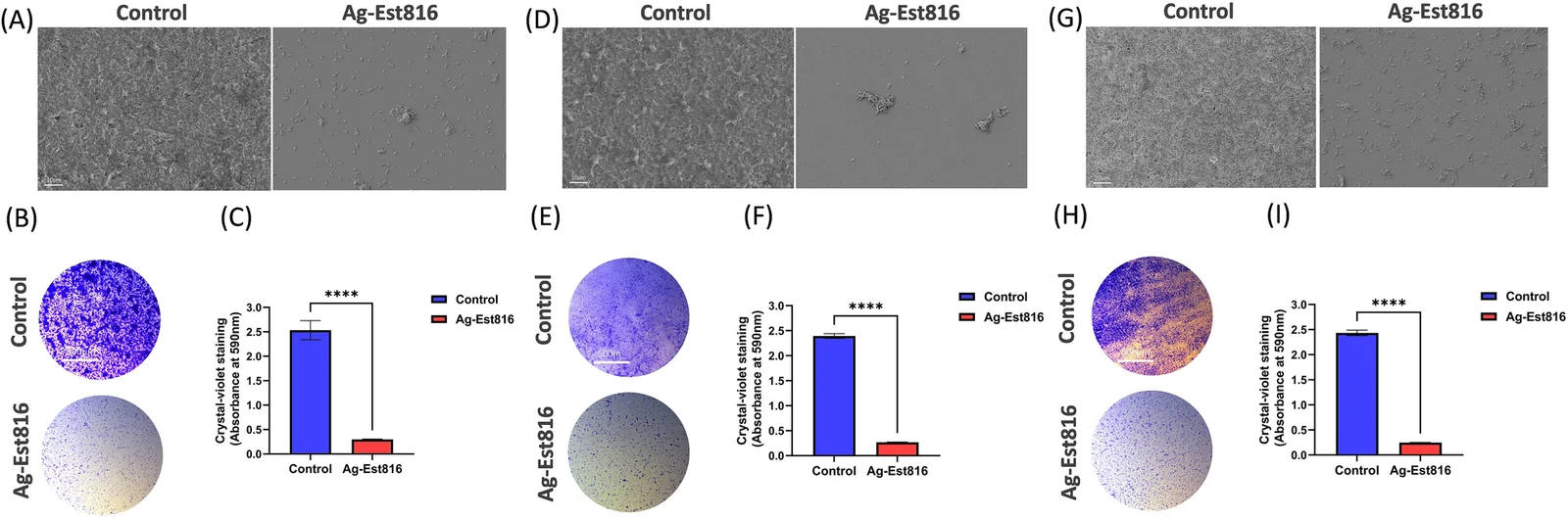
Effect of composite on biofilm morphology and biomass. **(A,D,G)** SEM images of the biofilms from Sample Nos. 7, 19, and 21, respectively, all at 1,000× magnification. **(B,E,H)** Image of the biofilm from Sample Nos. 7, 19, and 21, respectively, stained with crystal violet at 20× magnification. **(C,F,I)** Quantification of biofilm biomass at OD590 for Sample Nos. 7, 19, and 21.

## 4 Discussion

Quorum sensing allows bacteria to coordinate collective behaviors, with AHLs acting as key signaling molecules in Gram-negative bacteria, functioning in a density dependent manner (19). Variable AHLs have been detected in saliva and subgingival plaque from individuals. Beyond facilitating bacterial biofilm formation, AHLs also contribute to community structuring and promoting bacterial persistence within complex biofilm communities. Although AHL-based quorum sensing has traditionally been regarded as a hallmark of Gram-negative bacteria, evidence indicates that Gram-positive organisms can also sense and respond to these molecules (20). Our studies revealed that quorum sensing molecules facilitate integration into complex oral microbial communities. Disrupting AHL signaling altered subgingival biofilm composition, potentially influencing microbiome homeostasis and modulating the oral microbial ecosystem. We synthesized a silver nanocomposite integrated with the AHLs lactonase Est816 (Ag-Est816), which exhibits anti-biofilm property without causing dentin staining. In this study, we further investigated the effect of Ag-Est816 on supragingival plaque community.

The results showed that the Ag-Est816 composite significantly reduced the alpha and beta diversity metrics in this microbial community. In terms of alpha diversity, the Chao1, Observed species and Faith's PD index decreased significantly, while the Good's coverage index increased. Although Pielou's evenness showed a downward trend, it did not reach a statistical difference. It is suggested that under the current conditions, this material mainly reduces the species richness and phylogenetic diversity of the microbiota. In terms of beta diversity, PCoA based on weighted UniFrac distances showed a separation between the Control and Ag-Est816 groups. Subject-stratified PERMANOVA identified a statistically significant but modest difference in overall bacterial community composition between the two groups, indicating that treatment accounted for approximately 2.9% of the total community variation. PERMDISP analysis detected no statistically significant difference in within-group dispersion between the two groups. These findings suggest that Ag-Est816 treatment was associated with a modest but statistically significant shift in overall community composition, which was not attributable to differences in within-group dispersion (PERMDISP ns). The observed changes in community composition, together with the preservation of overall community evenness, suggest that Ag-Est816 may have the potential to modulate the ecological characteristics of ECC-associated biofilms while limiting extensive disruption of the microbial community. Furthermore, given the substantial inter-individual variation inherent in plaque-derived biofilm samples, the statistically significant PERMANOVA result indicates a detectable but modest treatment-associated difference in community composition. We hypothesise that a modest community-level shift may be compatible with a therapeutic strategy intended to modulate rather than extensively disrupt the oral microbiota.

At the phylum level, the composite decreased the relative abundance of *Actinobacteria*, whereas the relative abundance of *Firmicutes* increased. These changes may reflect an alteration in community composition following treatment, although the biological significance of these phylum-level shifts remains to be determined. *Actinobacteria* is higher in the dental plaque of children with ECC, which could adhere to the tooth surface and play a critical role in the initial formation of dental plaque (21). It contributes to the structural integrity and acidogenic environment of the biofilm, which may facilitate caries development. At the genus level, the Ag-Est816 composite significantly decreased the relative abundances of *Actinomyces* and *Lactobacillus* at the genus level*. Actinomyces* is considered a pioneer specie and contributor to the initiation of caries, serving as a substrate that facilitates colonization by primary cariogenic bacteria such as *Streptococcus mutans* (22). Therefore, the reduction in *Actinomyces* abundance suggests that the Ag-Est816 composite may help inhibit early biofilm formation and potentially reduce the risk of caries development in children with ECC. This microbial shift could reflect a microbial community restructuring toward a less cariogenic profile. Additionally, the metabolic products of *Lactobacillus*, such as lactic acid, promote enamel demineralization and facilitate the progression of dental caries (23). The decreased abundance of cariogenic bacteria, such as *Actinomyces* and *Lactobacillus*, suggests a dynamic microbial shift during ECC development. However, these findings contracts with those of Sikdar et al., who observed that AHLs lactonase increased the abundance of commensals and early colonizers, including *Actinomyces* (24)*.* The discrepancy may be due to differences in sample collection. Sikdar et al. used supragingival plaque from healthy individuals, whereas we collected plaque from children with ECC.

The clinical biofilm samples underwent *in vitro* amplification and multispecies biofilm cultivation prior to sequencing analysis, which may have altered the relative abundance of certain taxa compared with the native oral microbiota (25). Therefore, the microbial composition observed in the present study may not fully represent the original *in vivo* community structure. In addition, competitive interactions among different *Streptococcus* species under *in vitro* culture conditions may also have contributed to the relatively low abundance of *Streptococcus mutans* (26). These findings are consistent with the current ecological dysbiosis hypothesis, which suggests that dental caries is driven by complex shifts in microbial communities rather than by a single pathogen alone.

Notably, although the biofilms were cultured under aerobic conditions to better mimic the cariogenic environment associated with dental caries, 16S rRNA sequencing still detected anaerobic genera such as *Fusobacterium*, *Veillonella*, and *Prevotella*. The persistence of these taxa may be partly explained by the formation of oxygen gradients within the developing biofilms. Recent work by Sikdar et al. demonstrated that AHLs are produced and detectable in supragingival plaque-derived communities only under 5% CO_2_ conditions, whereas they are absent under strictly anaerobic conditions. Importantly, the same study further showed that this oxygen dependence applied to signal production rather than signal perception: although endogenous AHLs became undetectable under strictly anaerobic conditions, these communities remained responsive to exogenously supplied AHLs, which favoured late-colonizing taxa such as *Porphyromonas* (24). Likewise, von Ohle et al. demonstrated that in *in vitro* supragingival biofilms approximately 300 μm thick, regions deeper than 220 μm become anaerobic, with oxygen concentrations below 0.5% (27). Furthermore, many obligate anaerobes possess adaptive mechanisms that enable transient tolerance to oxygen exposure rather than being immediately eliminated upon aeration (28). Therefore, the detection of anaerobic-associated taxa in our aerobically cultured biofilms may reflect the development of oxygen-depleted microenvironments within the biofilm. However, this interpretation remains a possible explanation rather than direct evidence that the present ECC-derived enriched *in vitro* biofilm model reproduces the microbial composition or oxygen conditions of the *in vivo* oral environment.

These results suggest that Ag-Est816 treatment was associated with a restructuring of the bacterial community, characterized by changes in the relative abundance of several acid-producing and cariogenic taxa and a relative increase in some tolerant or opportunistic taxa. The treatment also altered the relative abundance of several taxa, with increases observed in *Haemophilus*, *Granulicatella*, and *Enterococcus*. Given the overall reduction in community richness following Ag-Est816 treatment, the increased relative abundance of these genera may partly reflect differential susceptibility among taxa and subsequent redistribution of ecological niches within the enriched biofilm community. In particular, taxa that were relatively less affected by the treatment may become proportionally more abundant following the reduction of susceptible members and associated interspecies competition. Therefore, the observed enrichment of *Haemophilus*, *Granulicatella*, and *Enterococcus* may represent relative community restructuring rather than a direct stimulatory effect of Ag-Est816 on these taxa. Further studies using absolute microbial quantification and taxon-specific assays are needed to determine whether these changes reflect actual increases in bacterial abundance.

Functional predictions based on PICRUSt2 indicated that the predicted abundance of gene families associated with starch and sucrose metabolism differed between the Ag-Est816 group and the Control group. Changes were also predicted in pathways related to amino acid and nucleotide sugar biosynthesis, suggesting potential differences in the functional capacity of the enriched biofilm community. Amino acid metabolism may play a role in stress responses and resistance mechanisms, serving as an adaptive strategy for bacteria under antimicrobial pressure (29). Moreover, the composite may lead to a decrease microbial diversity or shifts in community balance, affecting certain amino acid-synthesizing bacteria. In particular, nucleotide sugar biosynthesis is a key precursor process for extracellular polymeric substance (EPS) formation, a central factor in plaque biofilm development and drug resistance (30). Previous research has demonstrated that arginine exposure significantly downregulates nucleotide metabolism pathways in *Streptococcus mutans*, accompanied by marked reductions in both biofilm biomass and EPS production (31). Thus, the predicted changes in this pathway may be relevant to potential alterations in biofilm-associated functional capacity, although this interpretation requires direct experimental validation. Overall, PICRUSt2 analysis suggests that Ag-Est816 treatment was associated with changes in the predicted functional potential of enriched biofilm communities. However, these findings are inferred from 16S rRNA gene data and should not be interpreted as direct evidence of altered metabolic activity or gene expression.

SEM imaging and quantitative analysis of crystal violet staining demonstrated that Ag-Est816 composite markedly reduced biofilm biomass and disrupted the structural integrity of biofilms. This finding aligns with extensive existing evidence showing that AgNPs exert potent anti-biofilm effects through multiple mechanisms—such as inducing oxidative stress, interfering with EPS synthesis, and suppressing the expression of biofilm-related genes—thereby weakening bacterial adhesion and promoting biofilm disintegration (5, 32, 33). Meanwhile, the literature reports that silver nanoparticles have strong bactericidal activity and can limit the attachment of planktonic bacteria, further contributing to their anti-biofilm effect (34). By degrading signaling molecules that are essential for biofilm maturation and coordinated bacterial behavior, Est816 significantly inhibits multispecies biofilm formation, leading to structurally disintegrated architectures (35). Given that the two components were not separated in the experimental design, the reduction in biofilm biomass observed in this study is attributed to the Ag-Est816 composite as a whole. Quorum-quenching remains a plausible contributing mechanism: it has been reported to interfere with QS-regulated biofilm development (36). This mechanism is supported by previous work as a plausible contributor to the antibiofilm effect of the Ag-Est816 composite (16, 17, 35).

Although the present study focused on the antibiofilm activity and microbial ecological effects of Ag-Est816, its biosafety is also an important consideration for future clinical application. In our previous study, Ag-Est816 exhibited good cytocompatibility toward human gingival fibroblasts, indicating its potential biocompatibility as an oral antimicrobial material (17). Nevertheless, further *in vivo* biosafety evaluation remains necessary before clinical translation.

While the present study provides valuable insights into the effects of Ag-Est816 on cariogenic biofilms and microbial ecology, several limitations remain and warrant further investigation in future studies. While 16S rRNA sequencing primarily reflects structural changes in the microbiota, functional predictions require validation through metagenomics and metabolomics approaches. Second, the limited sample size restricts the ability to fully account for individual variations in microbiota composition. Future research should aim to verify the ecological effects and assess the long-term safety of the composite in animal models and clinical trials. Furthermore, a limitation of this study is that the efficiency of biofilm detachment was not directly quantified by microscopy. However, all samples were processed using an identical and strictly standardized protocol, which should minimize variability in biomass recovery and reduce potential bias across groups. Additionally, the individual contribution of AgNPs and the quorum-quenching enzyme Est816 within the Ag-Est816 nanocomposite was not separately evaluated. The primary aim of this work was to investigate the overall anti-biofilm and microbiome-modulating effects of the integrated nanocomposite against cariogenic multispecies biofilms. Previous studies have independently demonstrated the quorum-quenching effects of Est816 against oral pathogens (16). Future studies incorporating separate AgNPs and enzyme-only controls will help to better elucidate the underlying mechanisms of the Ag-Est816 nanocomposite system.

To address the low biomass obtained from children with ECC, a pre-culture step was applied to amplify the original plaque inocula to a standardized and workable level for reproducible biofilm formation and downstream molecular analyses. We acknowledge that this enrichment step does not preserve the original *in vivo* microbial community. It favors fast-growing, media-adapted taxa, particularly those capable of thriving in BHI medium. Consequently, the resulting biofilm model reflects a “complex oral polymicrobial biofilm” derived from the original plaque inocula rather than a direct replica of the *in situ* community. In future studies, we plan to employ saliva-supplemented SHI medium, which more closely mimics the nutritional conditions of supragingival plaque and better preserves the native microbial community.

Importantly, the primary aim of this *in vitro* model was to evaluate the comparative antibacterial and anti-biofilm efficacy of the Ag-Est816 composite under a controlled and reproducible experimental system. Notably, all experimental groups, including untreated controls and treatment conditions, were derived from the same enriched inoculum and subjected to identical handling and culture procedures. Thus, any potential bias introduced by the enrichment process was consistently applied across all groups, ensuring the validity of comparative analyses. Future research should further validate the ecological effects of the composite and evaluate its long-term safety in animal models and clinical trials.

## 5 Conclusion

In summary, the Ag-Est816 composite reduced overall bacterial richness and altered community composition, with reductions in several low-abundance cariogenic genera and changes in predicted microbial functions. It weakens the dominance of cariogenic bacteria and causes significant disruption to ECC-derived enriched *in vitro* biofilm model. The observed effects of the Ag-Est816 composite may be associated with the combined antibacterial and quorum-quenching properties of its components. However, the specific mechanisms underlying these effects require further investigation. Overall, our findings suggest that the Ag-Est816 composite has potential for modulating ECC-derived enriched *in vitro* biofilm model. This study provides new insights into potential therapeutic strategies for managing ECC.

## Data Availability

The raw 16S rRNA gene sequencing data generated in this study have been deposited in the NCBI Sequence Read Archive (SRA) under accession numbers SRR40427817–SRR40427876 and are publicly available in the NCBI SRA database. To ensure full reproducibility, the ASV abundance table (ASV×sample, with taxonomic annotations), sample metadata, R analysis scripts, and R session information (R version and package versions) have been deposited in Zenodo (DOI: 10.5281/zenodo.22685765).
