# Supplementary material for: N-acyl-homoserine lactonase–nanosilver composite modulates the bacterial community of an in vitro enriched biofilm model of early childhood caries
Source: Front Oral Health. 2026 Oct 2;7:1779148. doi: 10.3389/froh.2026.1779148 (PMC13671943; doi:10.3389/froh.2026.1779148)
Supplement: Supplementary file 1 [file Datasheet1.pdf]

1. Table S1. Per-sample sequencing depth and DADA2 quality-control summary. For each sample, the numbers of input, filtered, denoised, merged, and non-chimeric reads are reported, together with the final sequencing depth and observed ASV count. All 60 samples passed the sequencing quality-control criteria and were retained for downstream analysis.

Sample ID	Input	Filtered	Denoised	Merged	Non-chimeric	Sequencing Depth	Observed ASVs
C1C	108483	100505	99855	97533	80511	80490	275
C2C	79365	73656	73051	71003	57370	57355	247
C3C	100862	93911	93030	88910	64815	64752	454
C4C	97115	89063	87874	83208	56681	56599	612
C5C	93144	85672	84367	78894	58876	58764	587
C6C	73178	69721	69059	66571	43320	43282	345
C7C	62067	59588	58547	53387	31370	31193	643
C8C	82147	78518	77503	73133	48254	48177	600
C9C	77556	73545	73034	71409	50726	50714	275
C10C	84582	81109	80022	73975	48102	47936	772
C11C	79135	75544	74718	71311	48135	48055	433
C12C	83411	80136	79772	78164	59000	58975	218
C13C	87112	82170	81308	76963	58362	58281	581
C14C	93038	88709	88347	87274	66347	66340	225
C15C	77452	74188	73232	69702	46225	46169	420
C16C	79820	76617	76267	75117	57171	57158	242
C17C	72124	68253	67231	61361	40407	40276	759
C18C	88535	84113	83316	79011	58520	58448	506
C19C	72092	69011	68321	64476	45269	45178	497
C20C	85863	82332	81098	75884	54567	54454	551
C21C	88568	84547	83669	80073	58203	58139	356
C22C	80288	76257	75803	74000	59203	59183	264
C23C	74513	71214	70767	68712	50490	50466	248
C24C	67056	64088	63708	61844	44549	44532	308
C25C	72472	68759	68541	68004	62848	62843	112
C26C	92172	87648	87199	85440	63108	63082	231
C27C	89728	85962	85104	80499	62468	62380	584
C28C	96104	92358	91545	86896	63812	63728	546
C29C	95296	90413	89747	86505	64797	64766	357
C30C	85471	81859	80803	75255	57273	57178	675
C1E	93117	86474	85904	83734	67046	67025	278
C2E	79370	73711	73339	72077	60446	60439	190
C3E	99318	91112	90635	89002	72546	72530	249
C4E	99544	91266	90415	87756	73953	73928	436
C5E	101302	94157	93491	90020	72125	72096	403
C6E	78974	75424	75077	73820	59573	59562	280
C7E	76596	73205	72510	69508	53172	53132	507
C8E	89254	84438	84047	82144	63923	63908	237
C9E	112569	106798	106419	105336	92530	92520	239
C10E	89262	84772	83959	79426	54111	54032	650
C11E	99328	94919	94515	93017	74017	73999	284
C12E	88843	84179	83873	82575	67153	67143	164
C13E	77919	73762	72980	69401	53063	52969	596
C14E	88585	84734	84536	83578	72562	72559	161
C15E	86378	82515	81846	79459	60022	59996	272
C16E	99193	94270	93981	92989	82602	82593	229
C17E	91543	87408	86790	84067	65877	65847	397
C18E	86135	82135	81456	77983	59435	59386	437
C19E	79554	76145	75600	72703	56136	56083	366
C20E	108682	103103	102305	98211	75945	75889	450
C21E	110957	105059	104434	101452	77462	77435	288
C22E	102504	97506	97297	96398	77873	77868	112
C23E	53114	50769	50465	49326	41242	41238	211
C24E	98499	93101	92577	90721	73157	73136	297
C25E	84491	80187	79969	78907	63908	63897	111
C26E	95507	91206	91032	90268	77866	77864	108
C27E	96148	91904	91598	89495	72418	72403	266
C28E	101106	96309	95716	91020	66667	66602	452
C29E	112574	107375	106709	104136	80506	80489	283
C30E	111728	107283	106106	100667	73843	73736	762
